# News

**DOI:** 10.1155/S146392460100013X

**Published:** 2001


### News

### Gene Logic Inc. uses Staccato™ to generate gene expression data

Zymark corporation is pleased to announce that Gene Logic Inc. has chosen to use a Staccato Applications-Focused Workstation. Staccato's innovative liquid handling, stacking and control software technologies provide a uniform architecture using reliable and economical building blocks. Gene Logic will use the technology for multi-gene screening as part of its effort to generate the highest quality gene expression data.

'We are involved in a research collaboration focused on screening compounds against a large number of genes. We require a system that could be easily reconfigured as gene targets change or gene expression assays evolve', commented Eric Eastman, Senior Vice President and Chief Technology Officer for Gene Logic. 'Staccato will help us more efficiently measure the expression level of genes after the cells have been treated with compounds from a library. We are very excited to utilize this instrumentation in our multi-gene screening efforts for our custom discovery collaboration partner'.

'Our goal with Staccato is to provide scientists with a cost-efficient package that integrates the instruments most often used to accomplish specific goals in laboratory environments', stated Zymark Corporation President and CEO Kevin Hrusovsky. 'By carefully consolidating our offerings around what we know laboratories and scientists truly need, we are able to play a part in the challenge to get new drugs to market as quickly and cost efficiently as possible. With the Staccato technologies, Gene Logic is poised to accelerate their drug screening initiatives'.

Zymark Corporation's vision is to de-bottleneck the entire drug pipeline and be the premier provider of laboratory automation to the life science industry. The company is headquartered in Hopkinton, MA, and employs more than 365 people in the US, Canada, Europe and Japan. Zymark's Staccato Workstation is a scalable, cost-effective automated platform that expands throughput and capacity while supporting a range of different options, including high throughput screening, plate replication, cell based bioassays and automated hit picking procedures (for more information about Staccato and other Zymark products, please visit http://www.zymark.com).

*For additional information contact Jordana Estner, Marketing Communications, Tel:+ 1 (508)497-6541, e-mail: Jordana.Estner@zymark.com*

### Twister™ in MALDI-TOF and the Bruker Daltonics product line

Zymark Corporation of Hopkinton, MA, USA, has announced that Bruker Daltonics, Inc. has integrated Zymark's Twister Universal Microplate Handler with the new *autoflex*™ MALDI-TOF product. Twister is a low-cost, bench-top microplate handler designed to standardize laboratory applications for microplate readers, washers and liquid handlers. Bruker Daltonics is using Twister to feed MALDI microplate targets to the *autoflex* MALDI-TOF mass spectrometer. Twister provides an automated interface between the AnchorChip™ microplate format MALDI-targets while it can interface with a wide variety of instrumentation devices.

Bruker Daltonics is a leading developer and provider of innovative life science tools based on mass spectrometry. Its substantial investment in research and development allows it to design, manufacture and market a broad array of products intended to meet the rapidly growing needs of a diverse customer base, including pharmaceutical companies, biotechnology companies, proteomics companies, molecular diagnostics companies, academic institutions and government agencies. Bruker Daltonics has diverse technology platforms that integrate automated sample preparation and clean-up, advanced front-end AnchorChip MALDI targets and API source technology with cutting-edge proprietary MALDI-TOF, ESI-TOF, ion-trap and FTMS mass analysers, as well as analysis and bio-informatics software. It is also a worldwide leader in supplying mass spectrometry-based systems for substance detection and pathogen identification in security and defence applications.

*For more information contact Jordana Estner, Marketing Communications, Zymark Corporation of Hopkinton, MA, USA. Tel: + 1 (0)508 497 6549; e-mail: lynda.thomas@zymark.com; URL—http://www.zymark.com; or Bruker Daltonics. Tel: + 1 (0)978 663 3660, ext. 1149; URL—http://www.daltonics.bruker.com*

*Journal of Automated Methods & Management in Chemistry* ISSN 1463–9246 print/ISSN 1464–5068 online © 2001 Taylor & Francis Ltd
http://www.tandf.co.uk/journals
DOI: 10.1080/14639240110054109

